# The Potential Biomarker Panels for Identification of Major Depressive Disorder (MDD) Patients with and without Early Life Stress (ELS) by Metabonomic Analysis

**DOI:** 10.1371/journal.pone.0097479

**Published:** 2014-05-28

**Authors:** Xinghua Ding, Shuguang Yang, Wuju Li, Yong Liu, Zhiguo Li, Yan Zhang, Lingjiang Li, Shaojun Liu

**Affiliations:** 1 State Key Laboratory of Proteomics and Department of Neurobiology, Department of Neurobiology, Institute of Basic Medical Sciences, Beijing, China; 2 Center of Computational Biology, Institute of Basic Medical Sciences, Beijing, China; 3 Mental Health Institute, The Second Xiangya Hospital, Central South University, Changsha, Hunan, China; University of Electronic Science and Technology of China, China

## Abstract

**Objective:**

The lack of the disease biomarker to support objective laboratory tests still constitutes a bottleneck in the clinical diagnosis and evaluation of major depressive disorder (MDD) and its subtypes. We used metabonomic techniques to screen the diagnostic biomarker panels from the plasma of MDD patients with and without early life stress (ELS) experience.

**Methods:**

Plasma samples were collected from 25 healthy adults and 46 patients with MDD, including 23 patients with ELS and 23 patients without ELS. Furthermore, gas chromatography/mass spectrometry (GC/MS) coupled with multivariate statistical analysis was used to identify the differences in global plasma metabolites among the 3 groups.

**Results:**

The distinctive metabolic profiles exist either between healthy subjects and MDD patients or between the MDD patients with ELS experience (ELS/MDD patients) and the MDD patients without it (non-ELS/MDD patients), and some diagnostic panels of feature metabolites' combination have higher predictive potential than the diagnostic panels of differential metabolites.

**Conclusions:**

These findings in this study have high potential of being used as novel laboratory diagnostic tool for MDD patients and it with ELS or not in clinical application.

## Introduction

Major depressive disorder (MDD) is a serious psychiatric mood disorder, resulting in several detrimental socioeconomic effects, including increased healthy care expenditures and suicide rates [1]. And as a complex affective syndrome, the understanding of this disease is insufficient. Several well-established risk factors have been reported to increase an individual's likelihood of developing depression, including family history for depression, past personal history of depression, and early life stress (ELS) [2], [3]. There is study reported that the responsive of depressive disorder developed in relation to early life stress (ELS/MDD) to the treatment of depression is different [4]. So, the early life stress seems to be special in those risk factors. The epidemiological studies have provided strong evidence that the ELS, such as abuse, neglect or loss, is associated with dramatic increases in the risk to develop depression [5]–[7]. The studies in rodents and non-human primates reported that ELS induce persistent structural, functional, and epigenomic changes in some neural circuits. These changes converged in increased endocrine and autonomic reactivity to stress, anxiety-like behavior, anhedonia, cognitive impairment, pain sensitivity, and altered sleep [8]–[10]. Many of the neurobiological and behavioral effects of ELS in animal models closely parallel signs and symptoms of MDD. In addition, a group has conducted a series of clinical studies concerned whether early life adverse experience in humans is associated with neurobiological changes and whether the changes are related to depression. These studies focused on studying alterations of the HPA axis in subjects with histories of ELS and the results suggested that the ELS contributes to the neuroendocrine features of depression [4]. Because not all forms of depression are associated with ELS, the group reported the existence of biologically distinguishable subtypes of depression as a function of ELS [4]. Since the ELS/MDD may be a biologically distinguishable subtype of depression, one major question for clinical research concerned is whether there are characteristic alterations in human blood which can generate a detectable molecular phenotype for diagnoses.

Currently the firm diagnosis of the depressive disorder relies solely on the clinician's subjective identification of symptomatic clusters and scales which has the shortage of subjectivity [11]. Moreover, in routine practice, clinicians are typically challenged by fitting their patients' presentations, which lie along a continuous scale of depression severity, into strict DSM-IV based diagnostic categories, so over one-third of diagnosed depressed patients are not appropriately diagnosed [12]. An earlier clinical meta-analysis of 50371 depressed patients from 41 studies found the accuracy of symptom-based diagnosis of MDD to be a mere 47% [13]. In light of these factors, the development of empirical laboratory-based diagnostic approaches for MDD and its subtypes is required. Plasma is always chosen to be testing sample in the empirical laboratory-based diagnostic method for it can be collected at minimal risk and cost to the patients. And peripheral metabolic disturbances have been increasingly implicated in psychiatric mood disorder, including MDD [14]–[16]; it is therefore conceivable that introduction of metabonomic screen may generate a detectable molecular phenotype for diagnosis MDD and its subgroup (ELS/MDD and non-ELS/MDD) in plasma.

With the development of analytical technologies and methods, metabonomics approaches, which enables simultaneous quantitative measurement of numerous small molecules within a particular sample, are widely applied in the investigation of disease classification, potential biomarker discovery and molecular mechanism of diseases [17]–[19]. The metabolic profiling techniques, such as integrated gas chromatography/mass spectrometry (GC/MS) coupled with multivariate statistical analysis, are being widely used in metabonomics approaches [20]–[22]. Early studies employed the metabonomics approach have identified the panels of metabolites associated with depression-like behavior in animal models [23]–[25] and the metabolic perturbation in diagnosis of MDD patients [26], [27]. In this study, the central hypothesis was that there is characteristic metabolic alteration associated with the pathophysiologic mechanisms of the ELS/MDD in the blood which may generate a detectable molecular phenotype for diagnosis. Therefore, GC/MS coupled with multivariate statistical analyses was used to compare the metabolite profiles of plasma samples from ELS/MDD patients, non-ELS/MDD patients and healthy subjects. Furthermore, Tclass system [28]–[30], a machine learning method combining Fisher's linear discriminant analysis and feature selection based on a stepwise optimization process for classification and feature selection, was applied to overcome the positively biased cross-validation estimate induced by the diagnostic panels which was constituted by the pre-selecting differential metabolites [31] and to improve the predictive power of diagnostic metabolites' panels. The introduction of metabonomic screening and the Tclass system analysis may provide a novel empirical laboratory-based test for diagnosing MDD and its subtypes (ELS/MDD and non-ELS/MDD).

## Methods

### Subjects and sample collection

Plasma samples were collected from 25 healthy adults, 46 patients with chronic form of MDD, including 23 patients with previous ELS and 23 patients without previous ELS. The age ranges for above three groups were 27±5, 29±8, 30±6 years, respectively. All patients were diagnosed at the Second Xiangya Hospital of Central South University (Changsha, China). All subjects enrolled in this study volunteered to participate in this study. This study was approved by the Ethics Committee of the Second Xiangya Hospital of Central South University, China. A complete description of the study was provided to every subject and his or her legal guardians, and all participants had the capacity to consent. Written informed consent was obtained from each subject. All 71 subjects were examined for MDD according to the criteria of Diagnostic and Statistical Manual of Mental Disorders (DSM-IV) [32] and the 46 subjects were diagnosed as MDD patients. Severity of depression was measured with Self-Rating Depression Scale (SDS). The SDS was designed to assess the level of depression for patients diagnosed with MDD [33]. The Self-Rating Anxiety Scales (SAS) was used to monitor the anxiety mood in MDD patients. The SAS was only designed to measure the level of anxiety mood [34], [35]. The score on a rating scale, like SDS or SAS, is insufficient for diagnosing, and it just provides an indication of the severity of this symptom for a time period [36]. And then, the MDD patients were examined for childhood trauma. For assignment to the major depressive disorder patients with early life stress experience (ELS/MDD) group, the MDD patients must have had experienced at least one form of sexual or physical abuse before the age of 13 years. In our study, sexual abuse was defined as having been forced to touch another person's intimate parts, having been touched in intimate parts, attempted or completed intercourse. Physical abuse was defined as having been spanked, kicked or choked in a way that left bruises or injuries, having been attacked with a weapon or tied up or locked in a room or a closet. For assignment to the major depressive disorder patients without early life stress experience (non-ELS/MDD) group, the MDD patients could not have had experienced any traumatic or major stressful life event before the age of 13 years. The severity of the ELS was assessed by the Early Trauma Inventory (ETI). The ETI is a structured interview that assesses the number, frequency, and duration of early trauma types, resulting in a score for each trauma type and a total score [37], [38]. Blood samples were collected before breakfast on the second day after hospitalization with the EDTA-anticoagulant tube. After the centrifugation (3000×g) for 10 min at 4°C, the plasma samples were collected and stored at −80°C until analysis.

### Sample preparation

The pretreatment of plasma samples and GC/MS analysis were performed as previously described [39]–[41]. 500 µL of methanol (100%) and 20 µL of ribitol stock solution (0.2 mg/mL in deionized water) were added to 100 µL aliquots of thawed plasma samples. The mixture was shaken (100 rpm) at 70°C for 15 min and then centrifuged at 13000×g for 10 min. The supernatant was collected and mixed with chloroform (270 µL) and deionized water (450 µL). The mixture was shaken (80 rpm) at 37°C for 5 min and centrifuged at 4000×g for 10 min. The polar phase was separated and evaporated under a stream of N_2_ gas to dryness about 90 min. The dried residue was dissolved in 40 µL methoxamine hydrochloride (20 mg/mL pyridine) and incubated at 30°C for 90 min with continuous shaking. Then 40 µL of N-methyl-N-trimethylsilyl-trifluoroacetamide (MSTFA) containing 1% trimethylchlorosilane (TMCS) was added at 37°C for 30 min. The derivative samples were stored at room temperature for 120 min before injection. All chemicals were purchased from Sigma-Aldrich Chemical Co. (St. Louis, MI).

### GC/MS

0.3 µL aliquot of sample solution was injected at a split ratio of 25:1 into a GC/MS system consisting of a HP 6890 gas chromatograph and a time-of-flight mass spectrometer (Waters Co., Milford, MA). Chromatography was performed on a DB-5 MS capillary column (30 m×0.25 mm i.d., 0.25 mm thickness). Helium carrier gas was set to a constant flow rate of 1 mL/min. The temperatures of injection, interface, and ion source were adjusted at 230°C, 290°C, and 220°C, respectively, with an electron energy of 70 eV and a trap current of 70 µA. The GC oven temperature was first held at 70°C for 5 min solvent delay and then ramped at 5°C/min to a final temperature of 310°C, and this was followed by a 1 min isocratic, cool-down to 70°C, and an additional 5 min delay. The mass spectra over the m/z range 50–800 were acquired at a scan rate of 0.5 s per scan and an inter scan delay of 0.1 s in centroid mode. The GC/MS system was operated at a multichannel plate voltage of 2800 V, a pushout voltage of 980 V, and a pusher interval of 40 µs.

### Data processing and pattern recognition

Total ion current chromatograms (TICs) were obtained by using the MassLynx software (Waters Co.). Peaks with intensity higher than 10-fold of the signal-to-noise (S/N) ratio were recorded and integrated. The electron impact (EI) GC/MS data were converted into CDF files for peak extraction by Automated Mass Spectral Deconvolution and Identification System (AMDIS). The compounds in all recorded peaks in TICs were identified by using National Institute of Standards and Technology (NIST 02) library with EI spectra and then be validated using reference standards [42]. In addition, the GC/MS data were also processed using the MarkerLynx Applications Manager software (Waters Co.). A peak deconvolution package was incorporated in the software, which allowed the alignment of detection and retention times for peaks in each data file across the whole data set. MarkerLynx extracted components and generated a matrix of detected peaks that are represented by their m/z and retention time pairs along with their associated intensities. And intensities of the peaks of the validated compounds were normalized as relative peak area (RPA) to the ribitol's peak intensity which was defined as the internal standard, and the ribitol's peak intensity (internal standard intensity) was arbitrarily set to 1. The RPA was evaluated by multivariate data analysis (PCA and PLS-DA) to reduce the complexity of plasma GC data and facilitate analysis. The RPA data were also subjected to Tclass system for analysis.

### Discriminant analysis

To find out the biomarkers to discriminate patients from healthy subjects, the multivariate statistical analysis and the Tclass classification system were used in this study. For multivariate statistical analysis, PCA and PLS-DA were carried out for group discrimination. Based on the variable importance on a projection (VIP) with a threshold of 1.0 from the PLS-DA model, a number of metabolites variables were obtained to be responsible for the difference in the metabolic profiles between different groups, which were defined as the differential metabolites. And then, logistic regression was fit to find the diagnostic panel of differential metabolites between these groups.

In addition, Tclass classification system was applied to find out the diagnostic panel constituted by feature metabolites' combination [28]. At first, a feature forward selection procedure with the leave-one-out cross validation (LOOCV) as the object function was firstly applied to search for the optimal diagnostic panels, and then the stability index analysis was used to get an optimal biased assessment about how well the prediction model constructed by diagnostic panels will fit an independent data set. Through randomly dividing the sample into two parts with the partition ration 85% for 1000 times, the major part was used as the training set and the minor part was taken as the independent test set for each partition. The average of 1000 predictive accuracies from the test sets was defined as the stability index of the diagnostic panels which was suitable value for evaluating the performance of cross-validation estimates and the performance of predictive potential of the diagnostic panels in practice. Finally, the feature metabolites set with the highest stability indexes was found, and the related model that is composed of 1000 classifiers was constructed. Additionally, the ratio of the number of classifiers correctly predicting a sample and 1000 was taken as the probability (P) to predict MDD. Therefore, if P value is more than 0.5, the sample will be predicted to be MDD sample. The subgroups of MDD (ELS/MDD and non-ELS/MDD) were processed by the same analysis procedure.

Areas under the receiver operating characteristic curve (AUC) of the ROC analysis were calculated to evaluate the performance of these diagnostic panels. And these diagnostic biomarker panels were also validated by the Tclass system with the stability analysis.

## Results

### Demographic and clinical data

The demographic and clinical data were summarized in Table 1. There were no differences in age and racial distribution between different groups. Patients with MDD had significantly higher SDS score than that in healthy subjects [F (2,71) = 64.7, P<0.001]. And according to SAS score, the MDD patients had been observed existing anxiety mood [F (2,71) = 23.4, P = 0.004]. The 1990-92 National Comorbidity Survey (US) reported that 51% of those with MDD also suffer from lifetime anxiety [43]. And a study reported that the anxiety symptoms can have a major impact on the course of a depressive illness, with delayed recovery, increased risk of relapse, greater disability and increased risk of relapse, greater disability and increased suicide attempts [44]. Therefore, the anxiety mood can be often observed in the MDD patients. The MDD patients with a history of ELS had higher mean ETI score than those without ELS [F (1, 46) = 6.42, P<0.001]. There was no difference in current episode duration between two MDD groups (ELS/MDD group and non-ELS/MDD group). There was no difference in objective support between the two subgroups of MDD. Patients with MDD reported less subjective support and utilization of support than healthy subjects [F (2,71) = 3.71, P = 0.03] and [F (2,71) = 7.54, P = 0.001], respectively.

**Table 1:** Demographic and clinical features of healthy subjects, non-ELS/MDD and ELS/MDD.

	healthy subjects (n = 25)	non-ELS/MDD (n = 23)	ELS/MDD (n = 23)	Statistic
Age (mean, SD)^a^	27.5 (4.4)	29.8 (6.0)	29.2 (8.3)	F(2,71) = .80,ns
Race (n, %)				χ^2^(2) = 4.36, ns
the Han nationality	23 (92)	23 (100)	19 (82)	
other	2 (8)	0 (0)	4 (17)	
Education (mean, SD)^b^	17.3 (3.2)	11.4 (4.0)	13.8 (2.9)	F(2,71) = 17.6, P<0.001
Married or partnered (n, %)	8 (32)	13 (56)	11 (48)	χ^2^(2) = 3.09, ns
Employed (n, %)	25 (0)	16 (70)	18 (78)	χ^2^(2) = 7.24, P = 0.027
Length of current depression (mean, SD)^c^		21.7 (29.5)	27.1 (27.4)	F(1,46) = .00, ns
Family history of mental disorder (n, %)	0 (0)	2 (9.0)	3 (39)	χ^2^(2) = 3.14, ns
ETI Total (mean, SD)		33.3 (8.2)	49.2 (11.9)	F(1,46) = 6.42, P<0.001
Emotional abuse		6.6 (1.8)	9.7 (3.2)	F(1,46) = 8.38, P<0.001
Physical abuse		5.9 (1.7)	8 (3.1)	F(1,46) = 6.70, P = 0.007
Sexual abuse		5.4 (0.7)	6 (1.1)	F(1,46) = 3.1, P = 0.03
Emotional neglect		9.7 (1.9)	14.4 (4.6)	F(1,46) = 19.5, P<0.001
Physical neglect		7.2 (1.3)	11.1 (3.7)	F(1,46) = 17.5, P<0.001
SDS (mean, SD)	28.3 (5.3)	51.5 (11.0)	54.4 (8.3)	F(2,71) = 64.7, P<0.001
SAS (mean, SD)	26.6 (4.6)	42.3 (11.0)	42.7 (10.3)	F(2,71) = 23.4, P = 0.004
Social support (mean, SD)	37 (5.8)	32.2 (7.4)	29.5 (8.4)	F(2,71) = 6.10, P = 0.004
Objective support	8.4 (3.0)	7.4 (2.0)	7.1 (3.0)	F(2,71) = 1.44, ns
Subjective support	20.0 (3.8)	18.0 (5.1)	16.0 (5.6)	F(2,71) = 3.71, P = 0.03
Utilization of support	8.6 (1.6)	6.8 (2.1)	6.9 (1.6)	F(2,71) = 7.54, P = 0.001

Non ELS/MDD subjects have no history of childhood abuse; ELS/MDD subjects have history of childhood abuse. ETI, Early Trauma Inventory; SDS, Self-Rating Depression Scale; SAS, Self-Rating Anxiety Scale. a, in years; b, in years; c, in months.

### Metabolic profiles and differential metabolites of each group

**Figure 1:**
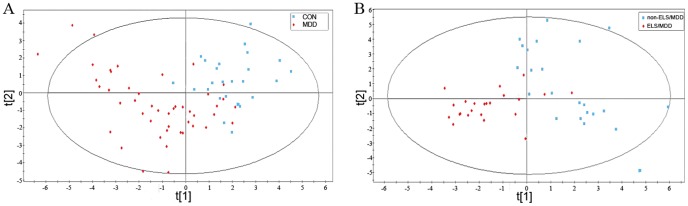
Score plots for PLS-DA of GC/TOF-MS data from healthy subjects, MDD patients and its subgroup (ELS/MDD patients and non-ELS/MDD patients). (A) The clustering analysis of metabonomic profiles from healthy subjects and MDD patients; (B) The clustering analysis of metabonomic profiles from ELS/MDD and non-ELS/MDD patients. MDD indicates depressed patients, ELS/MDD indicates depressed patients with early life stress experience and non-ELS/MDD indicates depressed patients without early life stress experience.

Typical GC/MS TICs of plasma samples from three groups were obtained. Thirty-five peaks of compounds were identified to be amino acids, fatty acids, carbohydrates, organic acids and mineral acid (Table 2). The results of the multivariate statistic analysis towards metabonomic data showed the distinct cluster between each group, indicating that the metabonomic data in each group have distinct metabolic profiles (Figure 1 and Figure S1). Through the PLS-DA loading plot (data not shown), many identified metabolites contributed strongly to the separation of groups were obtained. 15 metabolites stood out the VIP threshold (VIP>1), which were annotated to be differential metabolites between the healthy control and MDD (Table 2). In the 15 differential metabolites, 3 long-chain fatty acid (linoleic acid, oleic acid, heptadecylic acid) were found decreased in MDD patients; as for carbohydrate, the galactose and sorbitol were elevated while the myoinositol and mannose were decreased in MDD compared with healthy control; 4 amino acids (glycine, alanine, proline, serine) were found elevated while only leucine was decreased in MDD compared with healthy control. Erythronic acid was found decreased while butanedioic acid was found increased in MDD. Cholesterol was significantly decreased in MDD patients compared with healthy subjects. Through the same analysis procedure, 16 differential metabolites were annotated between the healthy control and ELS/MDD, including 6 amino acids (aspartic acid, glycine, alanine, threonine, serine, leucine); 5 carbohydrate (sorbitol, myoinositol, mannose, 6-deoxy-mannopyrannose, galactose); 3 long-chain fatty acid (linoleic acid, oleic acid, heptadecylic acid); 1 organic acid (erythronic acid) and cholesterol (Table 2). And then the 12 annotated differential metabolites, which were cholesterol, linoleic acid, glycine, alanine, butanedioic acid, lactic acid, glucose, oleic acid, glucopyranose, sorbitol, proline and stearic acid, were explored by PLS-DA model classifying the non-ELS/MDD and healthy control (Table 2). Moreover, the levels of cholesterol, glucopyranose, linoleic acid, glyceric acid, alanine, butanedioic acid, phosphoric acid, galactose, lactic acid, glycine, glucose, proline and stearic acid were identified relevant to the differentiation between the ELS/MDD and non-ELS/MDD (Table 2). These results suggested that some carbohydrates, amino acids and fatty acids contributed to the discrimination of ELS/MDD from healthy control or non-ELS/MDD.

**Table 2:** RPA of metabolites detected by GC/MS in human plasma^A^.

No.	Retention time (min)	Identified metabolites	Healthy subjects	MDD patients	ELS/MDD patients	non-ELS/MDD patients	VIP^B^
1	6.116	Lactic acid	1.6345±0.41723	1.9009±0.47168	1.7224±0.48594	2.0795±0.39025	VIP^c^ = 1.23 VIP^d^ = 1.17
2	7.033	Alanine	0.4348±0.07446	0.5018±0.13743	0.4365±0.09907	0.5672±0.14104	VIP^a^ = 1.31 VIP^b^ = 1.25 VIP^c^ = 1.33 VIP^d^ = 1.45
3	7.933	Oxalic acid	0.608±0.12699	0.6842±0.21615	0.6589±0.08991	0.7096±0.29350	
4	8.399	Hydroxybutyric acid	0.1686±0.16895	0.1484±0.168	0.1417±0.014071	0.1570±0.20161	
5	9.75	Valine	0.4496±0.06243	0.4517±0.1094	0.4410±0.06865	0.4624±0.13973	
6	10.866	Urea	3.1162±0.96887	3.0487±0.90941	2.9548±0.66486	3.1425±1.10959	
7	11.316	Phosphoric acid	3.4182±0.8181	3.6007±0.98405	3.1813±0.27462	4.0201±1.23997	VIP^d^ = 1.3
8	11.75	Leucine	0.17±0.04885	0.1661±0.0631	0.1535±0.04123	0.1805±0.08014	VIP^a^ = 1.03 VIP^b^ = 1.01
9	11.82	Proline	0.1108±0.07821	0.1788±0.1161	0.1359±0.07335	0.2162±0.1339	VIP^a^ = 1.05 VIP^c^ = 1.1 VIP^d^ = 1.05
10	12.05	Glycine	0.3351±0.07559	0.4490±0.1344	0.3990±0.07520	0.4990±0.16146	VIP^a^ = 1.38 VIP^b^ = 1.55 VIP^c^ = 1.39 VIP^d^ = 1.16
11	12.333	Butanedioic acid	0.0239±0.00882	0.0434±0.04028	0.0269±0.00782	0.0625±0.05293	VIP^a^ = 1.03 VIP^c^ = 1.24 VIP^d^ = 1.36
12	12.377	Glyceric acid	0.0204±0.01170	0.0214±0.01203	0.0162±0.00702	0.0285±0.01394	VIP^d^ = 1.5
13	13.5	Serine	0.1421±0.09798	0.2032±0.10309	0.1944±0.07278	0.2128±0.12977	VIP^a^ = 1.01 VIP^b^ = 1.13
14	14.15	Threonine	0.1622±0.10725	0.2205±0.11248	0.2144±0.08284	0.2269±0.13870	VIP^b^ = 1.15
15	16.233+17.767	Aspartic acid	0.9201±0.32851	1.1121±0.36793	1.1571±0.19671	1.0567±0.50531	VIP^b^ = 1.56
16	17.434	Pyroglutamate	0.1631±0.09368	0.1895±0.09589	0.1762±0.07038	0.2064±0.12116	
17	18.55	Erythronic acid	0.0260±0.02118	0.0168±0.00821	0.0169±0.00920	0.0167±0.007	VIP^a^ = 1.09 VIP^b^ = 1.05
18	23.951	Mannose	0.0482±0.02763	0.0355±0.02175	0.0307±0.02069	0.0414±0.2214	VIP^a^ = 1.02 VIP^b^ = 1.37
19	24.234	Isocitric acid	0.3288±0.13325	0.3388±0.10913	0.3373±0.05798	0.3410±0.15705	
20	24.867	Arabopyranose	0.8943±0.15508	0.9838±0.24707	0.9478±0.20382	1.0197±0.28393	
21	25.734	Unknown carbohydrate	1.6946±0.63606	1.8667±0.58277	1.7689±0.32809	2.0119±0.73246	
22	25.851	Glucose	3.7220±0.64182	4.2806±0.97574	3.9382±0.85764	4.6231±0.98319	VIP^c^ = 1.21 VIP^d^ = 1.08
23	26.201	Galactose	1.5240±0.17550	1.7945±0.34136	1.6611±0.32737	1.9278±0.30658	VIP^a^ = 1.32 VIP^b^ = 1 VIP^d^ = 1.19
24	26.434	Sorbitol	0.0175±0.0335	0.3028±0.63857	0.4385±0.819	0.1254±0.1759	VIP^a^ = 1.09 VIP^b^ = 1.44 VIP^c^ = 1.1
25	27.001	Gluconate	0.0805±0.04228	0.072±0.03247	0.0757±0.03107	0.0673±0.03449	
26	27.567	Glucopyranose	2.5494±0.42851	2.7061±0.59253	2.3710±0.40559	3.0413±0.56461	VIP^c^ = 1.16 VIP^d^ = 1.76
27	28.468	6-deoxy-mannopyrannose	0.1005±0.05662	0.1401±0.08907	0.1382±0.05129	0.1421±0.11898	VIP^b^ = 1.31
28	28.784	Palmitic acid	0.7542±0.19656	0.6426±0.19054	0.6827±0.17838	0.6024±0.19766	
29	29.534	Myoinositol	0.1330±0.05742	0.1041±0.05806	0.0989±0.02892	0.1098±0.07914	VIP^a^ = 1.1 VIP^b^ = 1.4
30	30.601	Heptadecylic acid	0.0169±0.00972	0.012±0.00565	0.0123±0.00544	0.0115±0.00641	VIP^a^ = 1.21 VIP^b^ = 1.15
31	31.484	Tryptophane	0.02±0.01	0.0232±0.01423	0.02±0.012	0.03±0.018	
32	31.751	Linoleic acid	0.1394±0.06166	0.0689±0.05385	0.0914±0.05650	0.0344±0.02334	VIP^a^ = 1.63 VIP^b^ = 1.49 VIP^c^ = 1.75 VIP^d^ = 1.53
33	31.868	Oleic acid	0.1874±0.09155	0.1102±0.0869	0.1292±0.07510	0.0836±0.09759	VIP^a^ = 1.3 VIP^b^ = 1.3 VIP^c^ = 1.21
34	32.384	Stearic acid	0.8864±0.18828	0.7775±0.2	0.8424±0.15551	0.7096±0.22150	VIP^c^ = 1.01 VIP^d^ = 1.01
35	45.652	Cholesterol	2.0814±0.4245	1.1613±0.91029	1.7824±0.66708	0.4711±0.58983	VIP^a^ = 1.6 VIP^b^ = 1.03 VIP^c^ = 2.18 VIP^d^ = 2.23

A , The normalized intensities of metabolites in healthy subjects, ELS/MDD patients and non-ELS/MDD patients are expressed with their RPA. Values are presented as mean±SD. ^B^VIP shows variable importance in the projection obtained from the PLS model with a cutoff of 1.0. a, shows variable importance in the projection obtained from the PLS model between the healthy subjects and MDD patients; b, shows variable importance in the projection obtained from the PLS model between the healthy subjects and ELS/MDD patients; c, shows variable importance in the projection obtained from the PLS model between the healthy subjects and non-ELS/MDD patients; d, shows variable importance in the projection obtained from the PLS model between the ELS/MDD patients and non-ELS/MDD patients.

### Diagnostic biomarker panels for identification of MDD and its subgroups (ELS/MDD and non-ELS/MDD)

Based on quantification of fewer metabolites, diagnosis will be more convenient and economical if the metabolites can provide sufficient information [31]. To explore the simplified and the optimal prediction diagnostic panels for MDD and even its subgroups (ELS/MDD and non-ELS/MDD), the ROC analysis and Tclass system were all applied.

At first, the differential metabolites in the plasma were used as biomarkers candidates. And then, the ROC analysis was carried out to find out the diagnostic panel from these biomarkers candidates. We sorted the VIP for each differential metabolite in a descending order. Logistic regression was then fit from 1 to 9 differential metabolites. According to the AUC of the ROC analysis, the logistic regression with 9 metabolites had the highest predictive potential because the AUC of this differential metabolites panel was 1 between the MDD and healthy control (Figure S2-A). According to the traditional academic scoring system, the AUC of 1 represents a perfect prediction test [31]. These 9 differential metabolites were linoleic acid, cholesterol, glycine, galactose, alanine, oleic acid, heptadecylic acid, myoinositol and sorbitol. Through the same analysis procedure, a logistic regression model with 9 differential metabolites was obtained, which had the highest predictive potential between ELS/MDD and healthy control. The AUC of this panel was 1 (Figure S2-B). These 9 differential metabolites were aspartic acid, glycine, linoleic acid, sorbitol, myoinositol, mannose, 6-deoxy-mannopyrannose, oleic acid and alanine. Between non-ELS/MDD and healthy control, the logistic regression with 3 metabolites was confirmed to have highest predictive potential, and the AUC of this panel was 1 (Figure S2-C). These 3 differential metabolites were cholesterol, linoleic acid and glycine. At last, a logistic regression model with 4 differential metabolites was found that have highest prediction potential between the ELS/MDD group and non-ELS/MDD group (Figure S2-D). These 4 differential metabolites were cholesterol, glucopyranose, linoleic acid and glyceric acid. The AUC of this panel was 1. However, Yang et al. reported that the pre-selecting differential metabolites may result in positively biased cross-validation estimates which will influence the prediction potential of the metabolic biomarkers panel [31]. Cross-validation estimate is an estimate value for assessing how accurately a prediction model will perform in practice. The traditional metabnomics studies have still used the pre-selecting differential metabolites to constitute the diagnostic panel for predicting diseases which will influence the predictive power of metabonomics approach. To overcome positively biased corss-validation estimates of the diagnostic panels, the Tclass system was applied which uses feature selection procedure such as stepwise optimization of all possible feature combinations and does not need the pre-selecting differential metabolites. And stability analysis was carried out to evaluate the performance of cross-validation estimates and the performance of predictive accuracy of each diagnostic panels obtained by Tclass system and the ROC analysis.

First of all, a model was generated by the Tclass system for classification between the healthy subjects and all MDD patients. The highest prediction power was reached through Naïve Bayes method with 9 metabolites combination, including valine, leucine, proline, glyceic acid, pyroglutamate, galactose, glucopyranose, palmitic acid and heptadecylic acid. The AUC of the feature metabolites' combination was 1 (Figure S2-E). Secondly, a model generated by Tclass system with 8 metabolites was obtained which had highest prediction power between the healthy control and ELS/MDD. And the AUC of these feature metabolites' combination including lactic acid, proline, glyceric acid, mannose, gluconate, tryptophane, stearic acid and cholesterol was 1 (Figure S2-F). Thirdly, a model with 3 metabolites (6-deoxidation mannopyrannose, palmitic acid and heptadecylic acid) was obtained which had the highest prediction power between the healthy control and non-ELS/MDD. The AUC of the feature metabolites' combination was also 1 (Figure S2-G). Finally, the optimal model generated by Tclass system with 3 metabolites (oxalic acid, heptadecylic acid and stearic acid) had the highest predictive power and the AUC of the feature metabolites' combination was 1 between the ELS/MDD and non-ELS/MDD (Figure S2-H).

### The cross-validation estimates evaluation of the diagnostic biomarker panels

To evaluate the cross-validation estimates and the prediction potential of each biomarker panels, the stability analysis was carried out. Through randomly dividing the sample into two parts with the partition ration 85% for 1000 times, the major part was used as the training set and the minor part was taken as the independent test set for each partition. The average of 1000 predictive accuracies from the test sets was defined as the stability index of the diagnostic panels which was suitable value for evaluating the performance of cross-validation estimates and the performance of predictive potential of the diagnostic panels in practice [28].

**Figure 2:**
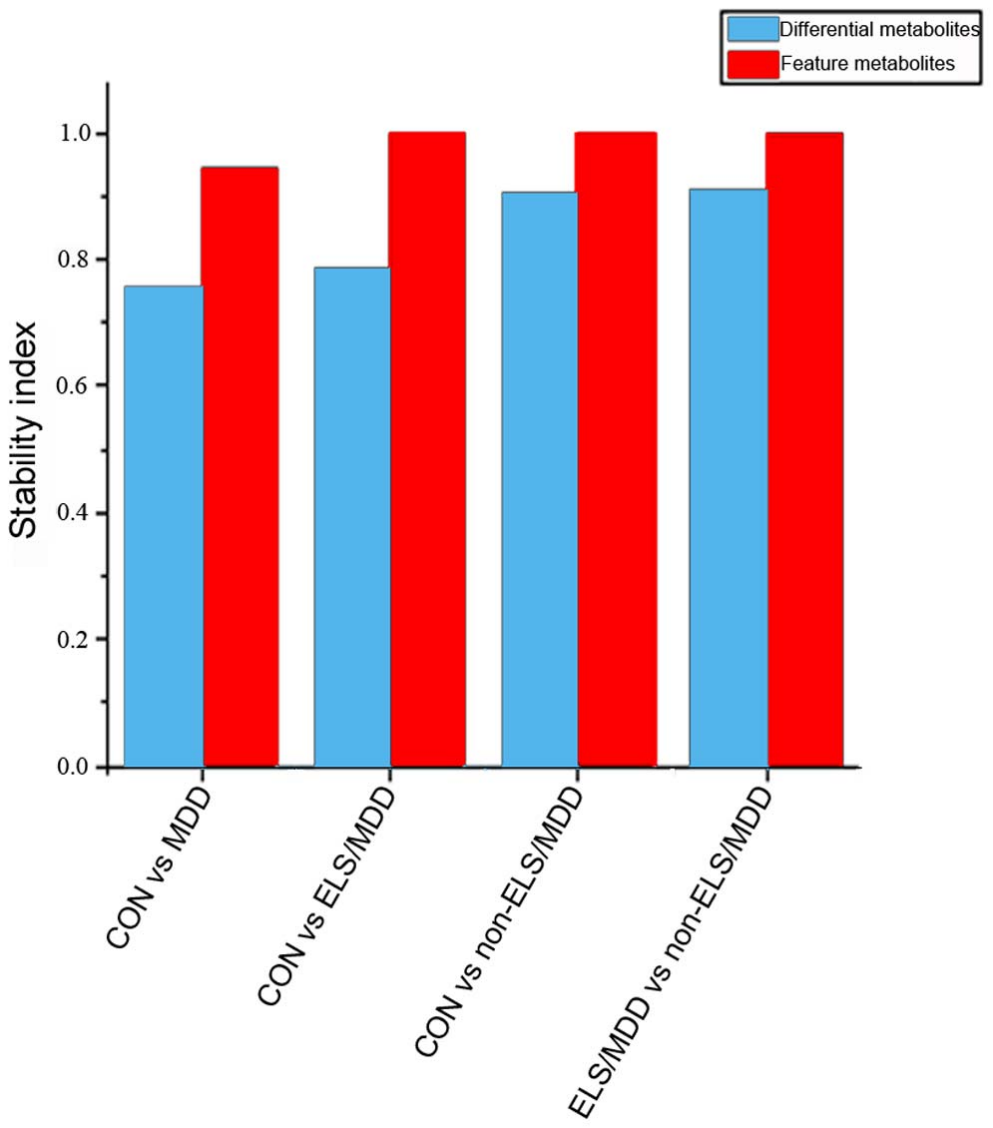
The stability index of each diagnostic panel. The diagnostic panels of feature metabolites' combination established by Tclass had the higher value than the corresponding diagnostic panels of differential metabolites, indicating the diagnostic panel of feature metabolites had higher predictive potential and more objective cross-validation estimate than the diagnostic panel of differential metabolites.

When the diagnostic panel of feature metabolites' combination obtained by Tclass system and the diagnostic panel of differential metabolites established by logistic regression of ROC analysis were both used to classify the healthy control and MDD, the stability index of the diagnostic panel constituted by differential metabolites was 0.7546 and the stability index of the diagnostic panel of feature metabolites' combination was 0.9438. When the stability index analysis analyzed the diagnostic panels between the healthy control and ELS/MDD, the stability index of the differential metabolites panel was 0.7872 and the stability index of the feature metabolites' set was 0.997. The stability index of the differential metabolites panel was 0.904 and the stability index of the feature metabolites' combination was 1 between the healthy control and non-ELS/MDD. At last, the stability index of differential metabolites panel was 0.9098 and the feature metabolites' combination was 1 between the ELS/MDD group and non-ELS/MDD group (Figure 2).

### The ensemble classifier model for identification MDD and its subgroups (ELS/MDD and non-ELS/MDD)

Here, the models generated by Tclass system were used for prediction disease. The relationship between the stability index and the number of feature metabolites for classification between the healthy subjects and all MDD patients was provided in Figure S3-A. The highest predictive power (prediction accuracy and stability index) was reached using Naïve Bayes method and the prediction accuracy through Tclass system was 97.436% at a sensitivity of 95.238% and a specificity of 100%. The final model constructed by the Tclass system was an ensemble classifier consisting of 1000 classifiers. The results were shown in Table S1. Here is the example of the one of the classifier during the ensemble classifier, which can objectively help for MDD identification.

C_1_ = -193.22739-348.65282X_1_+637.70420X_2_+37.96944X_3_+771.28313X_4_-179.95208X_5_-21.87057 X_6_+82.37111 X_7_+281.58456 X_8_+3245.21105X_9_

MDD_1_ = -143.65828-251.96156X_1_+453.25011X_2_+31.32127X_3_+475.62189X_4_-117.87757X_5_-10.38482X_6_+68.85657 X_7_+224.79745X_8_+2492.61184X_9_

The X_1_ (valine), X_2_ (leucine), X_3_ (proline), X_4_ (glyceic acid), X_5_ (pyroglutamate), X_6_ (galactose), X_7_ (glucopyranose), X_8_ (palmitic acid) and X_9_ (heptadecylic acid) stand for the RPA level of metabolites in the diagnostic panel. For one plasma sample, the RPA values for these metabolites were applied to the 1000 classifiers like this and the sample will be predicted to be the sample of MDD patients if there were more than 500 classifiers in which the value in the equation of “MDD” is bigger than the value in the equation of “C”. The relationship between the predictive power and the number of feature metabolites for classification between the healthy subjects group and ELS/MDD patients group was displayed in Figure S3-B. The prediction accuracy through Tclass system was 100% (Sensitivity is 100%; Specificity is 100%), when combining eight metabolites. 1000 related classifiers were taken as the final classification profile and the results were shown in Table S1. The relationship between the predictive power and the number of feature metabolites for classification between the healthy subjects and non-ELS/MDD patients was provided in Figure S3-C. The optimal prediction result was obtained from the combination of 3 metabolites. The prediction accuracy was as high as 100% (Sensitivity is 100%; Specificity is 100%). The related classifiers were displayed in Table S1. Finally, the Tclass system was applied to generate a model for classification between the ELS/MDD and non-ELS/MDD groups. The relationship between the predictive performance and the number of metabolites was provided in Figure S3-D. The optimal prediction result was obtained from the combination of 3 metabolites and their prediction accuracy was 100% (Sensitivity is 100%; Specificity is 100%). The related classifiers were shown in Table S1. These models established by the Tclass system could be used to identify MDD and its subgroups.

## Discussion

MDD is a serious psychiatric mood disorder and it is also a complex affective syndrome. Numerous epidemiologic and clinical studies have provided compelling evidence for a strong association between various forms of early life stress and depressive symptoms or disorders [5], [45], [46]. Recently, several studies reported that the depression developed in relation to early life stress experiences (ELS/MDD) have a characteristic neuroendocrine alterations associated with the pathophysiologic mechanisms of ELS/MDD [4]. To explore the characteristic metabolic alterations of ELS/MDD, we developed a metabonomics approach that uses GC/MS coupled with multivariate statistical analysis for identifying the differences in the global plasma metabolites and generating mathematic models for diagnosis. Our approach included 3 steps: 1) the distinct metabolic profile of the MDD and its subgroups (ELS/MDD and non-ELS/MDD) exploration; 2) diagnostic biomarker panels for prediction of MDD patients and its subgroups (ELS/MDD and non-ELS/MDD) investigation; 3) the diagnostic models for prediction of MDD patients and its subgroups (ELS/MDD and non-ELS/MDD) construction.

### Limitations

Through the multivariate statistical analysis, we found that it is possible to separate the MDD and its subgroup (ELS/MDD and non-ELS/MDD) with the plasma metabonomic data. The results of the analysis indicated that each group has distinct metabolic profiles in the blood (Figure 1 and Figure S1). So the aim of this study was to examine the feasibility of an empirical laboratory-based method to diagnose MDD and even its subgroup (ELS/MDD and non-ELS/MDD). In this study, plasma samples were collected from 25 healthy subjects, 46 patients with MDD, including 23 patients with ELS and 23 patients without ELS. We made appropriate refinement towards the traditional metabonomic data analysis approach to obtain the mathematic model with optimal predictive power from the relatively small sample size. In the most of the metabonomics studies, the diagnostic biomarkers panels for prediction diseases were constituted by the pre-selecting differential metabolites. However, the pre-selecting differential metabolites may result in positively biased cross-validation estimates which will influence the predictive power of the metabonomics approach [31]. In this metabonomics study, the Tclass system was applied to search the optimal feature combinations of metabolites for diagnosis and prognosis. Take the advantages of selecting biomarker panel without needing pre-selecting differential metabolites, the Tclass system can overcome the positively biased cross-validation estimates and improve the predictive power of the metabonomics method [28]. Through application of Tclass system, the GC/MS based plasma metabonomics approach identified MDD patients at a sensitivity of 95.2381% and a specificity of 100%; and identified its subgroups, ELS/MDD patients, at a sensitivity of 100% and specificity of 100%. The small size of the training set for the prediction model required achieving greater than 90% sensitivity and specificity highlights the power of this approach [47]. Our analysis is preliminary, and substantially larger sample size obtained through application of this refined metabonomics approach to clinical practice may further improve the diagnostic sensitivity and specificity of this novel metabonomics approach.

### Novel metabonomic insights and diagnostic method about ELS/MDD

The results of multivariate statistic analysis suggested that the ELS/MDD have characteristic metabolic alterations associated with the pathophysiologic mechanisms of ELS/MDD in the blood. The PLS-DA model implicated 16 metabolites responsible for discrimination ELS/MDD and healthy control, and 13 metabolites responsible for discrimination ELS/MDD and non-ELS/MDD. The overlapping metabolites from these two metabolites sets may play a role in ELS/MDD pathophysiologic mechanism and in discriminating ELS/MDD from MDD. These metabolites included amino acids (alanine, glycine), carbohydrate (galactose), fatty acids (linoleic acid), and cholesterol.

The altered amino acid profile was noteworthy in light of previous studies that suggested the synthesis of brain neuro-transmitters related to ELS/MDD pathophysiologic mechanism can be influenced by circulating amino acids levels [27], [48], [49].The previous study reported that the higher plasma levels of alanine, glutamine, glycine, and taurine are existed in MDD patients [50] and our study also found the higher plasma levels of alanine and glycine in MDD including ELS/MDD and non-ELS/MDD. In this study, the plasma levels of alanine and glycine were decreased in the ELS/MDD when they were compared to non-ELS/MDD. These findings implicated that the decreased plasma levels of alanine and glycine may be associated with the ELS/MDD mechanism and provide an ELS-associated metabolic alteration in discriminating ELS/MDD from non-ELS/MDD. The plasma level of galactose was found elevated in MDD compared with healthy control. In MDD, the level of galactose in ELS/MDD was lower than non-ELS/MDD. Galactose is an important metabolites involved in the formation of glycans featuring galactose and its derivatives which were essentially bound by galectins [51], [52]. Converging evidence suggested that galectins play an important role in neuroinflammation and brain development and function [53]–[55], and Kraneveld et al. reported that dietary or pharmacological modulation with small molecules targeting the galectin response in neurodevelopment disorders such as MDD could be a future therapeutic approach [56]. Although galactose's role in MDD is not clear, the foregoing reports implied that galactose may be involved in galectin-glycan interactions associated with the neuro-immune axis in mental disorders such as MDD and ELS/MDD. Linoleic acid level in the plasma was found decreased in MDD (including ELS/MDD and non-ELS/MDD) compared to healthy control. Inside the MDD group, the plasma level of linoleic acid in ELS/MDD patients was higher than the MDD patients without ELS. Consistent with these findings, a previous study reported that the plasma level of linoleic acid in MDD patients was significantly lower than the level of healthy subjects [57]. And many studies reported that ELS altered the metabolic profile of plasma polyunsaturated fatty acids in adulthood [58], [59]. Even a study reported that dietary n-3 polyunsaturated fatty acid, such as linoleic acid, deprivation together with early maternal separation increased anxiety and vulnerability to stress in adult rats [60]. These findings indicated that higher plasma level of the linoleic acid in ELS/MDD compared with non-ELS/MDD may be an ELS-associated metabolic alteration in depression patients. And this study also found the lower plasma level of cholesterol in MDD (including ELS/MDD and non-ELS/MDD) and this finding was consistent with the several previous reports' findings [61], [62]. The plasma level of cholesterol in ELS/MDD was significantly higher than the level of non-ELS/MDD. A previous study also reported that the early-life maltreatment may induce high level of cholesterol in the adulthood of non-human primate [63]. Those ELS-associated metabolic alterations indicated the potential of plasma metabonomics method in discriminating ELS/MDD from MDD and provided a novel plasma metabolic insight about the ELS/MDD.

Due to the lack of empirical laboratory-based tests, the diagnosis of MDD relies solely on the clinician's subjective identification of symptomatic clusters and scales which has the shortage of subjectivity [11]. The lack of the disease molecular markers to support objective laboratory tests constitutes a bottleneck for the research on MDD. In light of this shortage, this study applied ROC analysis and Tclass system to obtain the metabolic biomarker panels for predicting MDD and even its subgroup (ELS/MDD and non-ELS/MDD). Through the ROC analysis, the diagnostic panels with pre-selecting differential metabolites were obtained. The AUC of these obtained diagnostic panels all attained 1. To overcome the positively biased cross-validation estimate of the differential metabolites' panel, the Tclass system was applied which does not need the pre-selecting differential metabolites to constitute the diagnostic panels and construct models [28], [31]. The diagnostic panels with feature metabolites' combination were obtained by Tcalss system analysis. The AUC values of these diagnostic panels all attained 1, as well. To evaluate the cross-validation estimates of these diagnostic panels obtained by Tclass system analysis or ROC analysis, the stability analysis was carried out. The stability index of stability analysis was a suitable cross-validation value for evaluating the performance of cross-validation estimates and the performance of predictive potential of the diagnostic panels in practice. Our preliminary results showed that the stability index of the feature metabolites' combination was generally higher than the differential metabolites panel (Figure 2). When using the diagnostic panels, for instance, classify the healthy control and MDD, the stability index of the diagnostic panel constituted by differential metabolites was 0.7546 and the stability index of the diagnostic panel constituted by feature metabolites' combination was 0.9438. Therefore, the feature metabolites' combination obtained by Tclass system had the optimum biased cross-validation estimate and had more accurate predictive potential compared with the diagnostic panels of differential metabolites obtained by ROC analysis.

Accordingly, 4 mathematical models (Table S1) were generated by Tclass system and these models can be used for prediction MDD and its subgroup (ELS/MDD and non-ELS/MDD). And on the basis of these mathematical models, the 3 prediction tools were designed including the prediction tool for MDD, ELS/MDD, and non-ELS/MDD (Table S1). At first, we should use the prediction tool for MDD to identify the sample from MDD or not. The ensemble classifier model for healthy control and MDD is applied. And if the sample is predicted as MDD, the sample is from a MDD patient. Next, we will use the prediction tool for ELS/MDD to identify the sample from ELS/MDD or not. When using the prediction tool for diagnosing ELS/MDD, we actually apply 2 ensemble classifier models (healthy control vs. ELS/MDD and ELS/MDD vs. non-ELS/MDD). Only when the patient's plasma sample has been both predicted as ELS/MDD by the 2 ensemble classifier model (healthy control vs. ELS/MDD and ELS/MDD vs. non-ELS/MDD), the patient will be predicted as ELS/MDD. In consistent with it, only when the plasma sample has been both predicted as non-ELS/MDD by both 2 models (healthy control vs. non-ELS/MDD and ELS/MDD vs. non-ELS/MDD), the plasma will be identified from non-ELS/MDD. These tools were shown in Table S1. In these prediction tools, what the user needs to do is to extract the RPA of the feature metabolites that constitute the diagnostic panel with highest classification accuracy. Then, paste the above RPA of feature metabolites into the metabolites window. The related discrimination result will be displayed in the discrimination window.

In summary, this study used metabonomics approach based on GC/MS coupled with multivariate statistic analysis to characterize the metabolic profiles of plasma from MDD and its subgroups (ELS/MDD and non-ELS/MDD). The results showed that the subgroup of MDD patients with ELS have the distinct metabolic profiles when compared to non-ELS/MDD patients and healthy subjects. And the diagnostic panels of feature metabolites' combination and the ensemble classifiers provide a novel metabonomics approach for diagnosis and prognosis of MDD even its subgroups (ELS/MDD and non-ELS/MDD) which can improve the predictive power of the biomarker panels obtained by the current metabonomic data analysis approach. Although the introduction of metabonomic screening and the Tclass system analysis can help to simply and objectively diagnose MDD and even its subgroups (ELS/MDD and non-ELS/MDD); the limitations in this study indicated that the further studies with large sample size are required to replicate and validate this novel metabonomic analysis approach.

## Supporting Information

Figure S1 **Score plots for PLS-DA of GC/TOF-MS data from healthy subjects, ELS/MDD patients and non-ELS/MDD patients.** The clustering analysis of metabonomic profiles from healthy subjects and ELS/MDD patients (A); the clustering analysis of metabonomic profiles from healthy subjects and non-ELS/MDD patients (B). MDD indicates depressed patients, ELS/MDD indicates depressed patients with early life stress experience and non-ELS/MDD indicates depressed patients without early life stress experience.
(DOCX)

Figure S2 **Scatter plots of the values of area under the receiver operating characteristic curve (AUC) of ROC analyses.** The Scatter plots of the values of AUC were drawn for evaluating the diagnostic panels of differential metabolites between the healthy subjects and MDD patients (A), healthy subjects and ELS/MDD patients (B), healthy subjects and non-ELS/MDD patients (C), ELS/MDD patients and non-ELS/MDD patients (D); and the results of ROC analyses for evaluating the diagnostic panels of feature metabolites' combination between healthy subjects and MDD patients (E), healthy subjects and ELS/MDD patients (F), healthy subject and non-ELS/MDD patients (G), ELS/MDD patients and non-ELS/MDD patients (H). The relationship between the number of metabolites and the diagnostic performance was shown by the AUC values which were based on the receiver operating characteristic (ROC) analysis and logistic regression model analysis.
(DOCX)

Figure S3 **The results of Tclass discriminant analyses.** Results of Tclass discriminant analyses between the healthy subjects and MDD patients (A), healthy subjects and ELS/MDD patients (B), healthy subjects and non-ELS/MDD patients (C), ELS/MDD patients and non-ELS/MDD patients (D). The relationship between the number of metabolites and classification accuracy was shown by Fisher's test and Naïve Bayes discriminant analysis. Both methods were based on the feature forward selection procedure and classification accuracy from leave-one-out cross-validation (LOOCV).
(DOCX)

Table S1 The ensemble classifier models and the prediction tools for identification MDD and its subgroups (ELS/MDD and non-ELS/MDD). This is an excel file with 7 worksheets. “Ensemble classifier model 1” worksheet describes 1000 classifiers which consist of the ensemble classifier model for discrimination healthy control and MDD; “Ensemble classifier model 2” worksheet describes the ensemble classifier model for discrimination healthy control and ELS/MDD; “Ensemble classifier model 3” worksheet describes the ensemble classifier model for discrimination healthy control and non-ELS/MDD; “Ensemble classifier model 4” worksheet describes the ensemble classifier model for discrimination non-ELS/MDD and ELS/MDD. And then, “Prediction tool for MDD” worksheet describes a prediction tool to identify the sample from MDD or not; “Prediction tool for ELSMDD” worksheet describes the prediction tool to identify the sample from ELS/MDD or not; “Prediction tool for non ELSMDD” worksheet describes the prediction tool to identify the sample from non-ELS/MDD or not. In these prediction tools, what the user needs to do is to paste the RPA of the feature metabolites into the metabolites window, and then the related discrimination result will be displayed in the discrimination window.
(XLSX)
