# Supplementary material for: The Potential Biomarker Panels for Identification of Major Depressive Disorder (MDD) Patients with and without Early Life Stress (ELS) by Metabonomic Analysis
Source: PLoS One. 2014 May 28;9(5):e97479. doi: 10.1371/journal.pone.0097479 (PMC4037179; doi:10.1371/journal.pone.0097479)
Supplement: Figure S1 — Score plots for PLS-DA of GC/TOF-MS data from healthy subjects, ELS/MDD patients and non-ELS/MDD patients. The clustering analysis of metabonomic profiles from healthy subjects and ELS/MDD patients (A); the clustering analysis of metabonomic profiles from healthy subjects and non-ELS/MDD patients (B). MDD indicates depressed patients, ELS/MDD indicates depressed patients with early life stress experience and non-ELS/MDD indicates depressed patients without early life stress experience. (DOCX) [file pone.0097479.s001.docx]

**
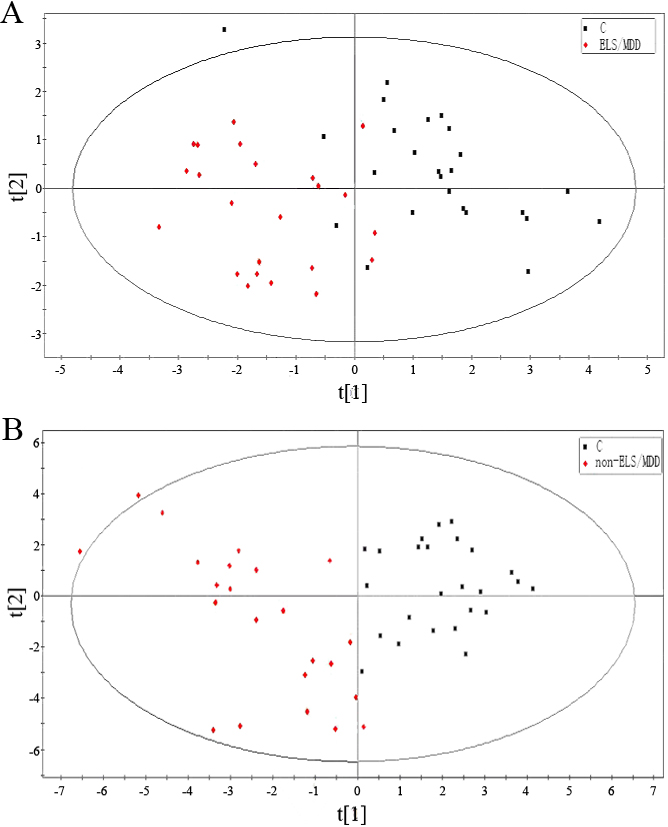
Figure S1. Score plots for PLS-DA of GC/TOF-MS data from healthy subjects, ELS/MDD patients and non-ELS/MDD patients.** The clustering analysis of metabonomic profiles from healthy subjects and ELS/MDD patients (A); the clustering analysis of metabonomic profiles from healthy subjects and non-ELS/MDD patients (B). MDD indicates depressed patients, ELS/MDD indicates depressed patients with early life stress experience and non-ELS/MDD indicates depressed patients without early life stress experience.
