# Supplementary material for: The Potential Biomarker Panels for Identification of Major Depressive Disorder (MDD) Patients with and without Early Life Stress (ELS) by Metabonomic Analysis
Source: PLoS One. 2014 May 28;9(5):e97479. doi: 10.1371/journal.pone.0097479 (PMC4037179; doi:10.1371/journal.pone.0097479)
Supplement: Figure S2 — Scatter plots of the values of area under the receiver operating characteristic curve (AUC) of ROC analyses. The Scatter plots of the values of AUC were drawn for evaluating the diagnostic panels of differential metabolites between the healthy subjects and MDD patients (A), healthy subjects and ELS/MDD patients (B), healthy subjects and non-ELS/MDD patients (C), ELS/MDD patients and non-ELS/MDD patients (D); and the results of ROC analyses for evaluating the diagnostic panels of feature metabolites' combination between healthy subjects and MDD patients (E), healthy subjects and ELS/MDD patients (F), healthy subject and non-ELS/MDD patients (G), ELS/MDD patients and non-ELS/MDD patients (H). The relationship between the number of metabolites and the diagnostic performance was shown by the AUC values which were based on the receiver operating characteristic (ROC) analysis and logistic regression model analysis. (DOCX) [file pone.0097479.s002.docx]

**
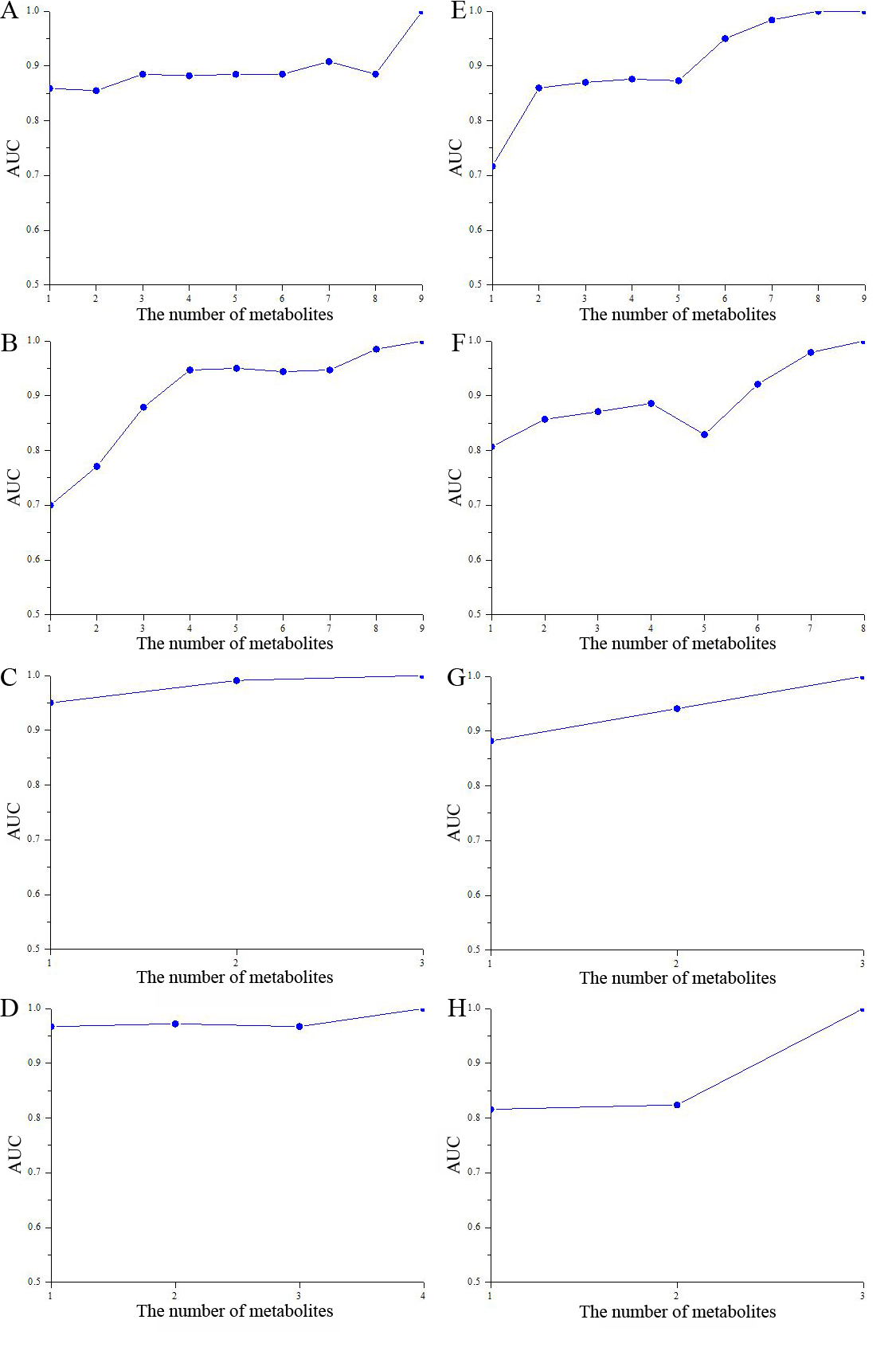
Figure S2. Scatter plots of the values of area under the receiver operating characteristic curve (AUC) of ROC analyses.** The Scatter plots of the values of AUC were drawn for evaluating the diagnostic panels of differential metabolites between the healthy subjects and MDD patients (A), healthy subjects and ELS/MDD patients (B), healthy subjects and non-ELS/MDD patients (C), ELS/MDD patients and non-ELS/MDD patients (D); and the results of ROC analyses for evaluating the diagnostic panels of feature metabolites’ combination between healthy subjects and MDD patients (E), healthy subjects and ELS/MDD patients (F), healthy subject and non-ELS/MDD patients (G), ELS/MDD patients and non-ELS/MDD patients (H). The relationship between the number of metabolites and the diagnostic performance was shown by the AUC values which were based on the receiver operating characteristic (ROC) analysis and logistic regression model analysis.
