# Supplementary material for: The Potential Biomarker Panels for Identification of Major Depressive Disorder (MDD) Patients with and without Early Life Stress (ELS) by Metabonomic Analysis
Source: PLoS One. 2014 May 28;9(5):e97479. doi: 10.1371/journal.pone.0097479 (PMC4037179; doi:10.1371/journal.pone.0097479)
Supplement: Figure S3 — The results of Tclass discriminant analyses. Results of Tclass discriminant analyses between the healthy subjects and MDD patients (A), healthy subjects and ELS/MDD patients (B), healthy subjects and non-ELS/MDD patients (C), ELS/MDD patients and non-ELS/MDD patients (D). The relationship between the number of metabolites and classification accuracy was shown by Fisher's test and Naïve Bayes discriminant analysis. Both methods were based on the feature forward selection procedure and classification accuracy from leave-one-out cross-validation (LOOCV). (DOCX) [file pone.0097479.s003.docx]

**
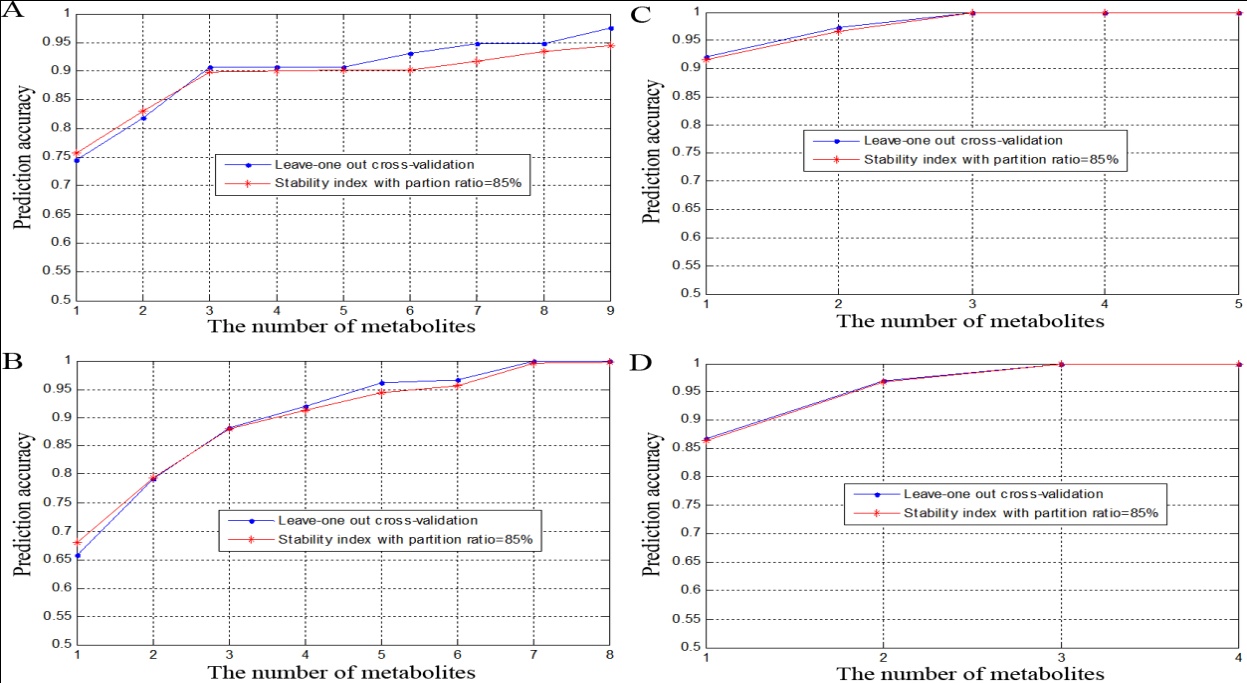
Figure S3. The results of Tclass discriminant analyses.** Results of Tclass discriminant analyses between the healthy subjects and MDD patients (A), healthy subjects and ELS/MDD patients (B), healthy subjects and non-ELS/MDD patients (C), ELS/MDD patients and non-ELS/MDD patients (D). The relationship between the number of metabolites and classification accuracy was shown by Fisher’s test and Naïve Bayes discriminant analysis. Both methods were based on the feature forward selection procedure and classification accuracy from leave-one-out cross-validation (LOOCV).
